# Nitrogen Fuelling of the Pelagic Food Web of the Tropical Atlantic

**DOI:** 10.1371/journal.pone.0131258

**Published:** 2015-06-22

**Authors:** Vera Sandel, Rainer Kiko, Peter Brandt, Marcus Dengler, Lars Stemmann, Pieter Vandromme, Ulrich Sommer, Helena Hauss

**Affiliations:** 1 GEOMAR Helmholtz Centre for Ocean Research Kiel, Düsternbrooker Weg 20, Kiel, Germany; 2 Sorbonne Universités, UPMC Univ Paris 06, UMR7093, LOV, Observatoire océanologique, Villefranche-sur-mer, France; CSIR- National institute of oceanography, INDIA

## Abstract

We estimated the relative contribution of atmosphere (ic Nitrogen (N) input (wet and dry deposition and N fixation) to the epipelagic food web by measuring N isotopes of different functional groups of epipelagic zooplankton along 23°W (17°N-4°S) and 18°N (20-24°W) in the Eastern Tropical Atlantic. Results were related to water column observations of nutrient distribution and vertical diffusive flux as well as colony abundance of *Trichodesmium* obtained with an Underwater Vision Profiler (UVP5). The thickness and depth of the nitracline and phosphocline proved to be significant predictors of zooplankton stable N isotope values. Atmospheric N input was highest (61% of total N) in the strongly stratified and oligotrophic region between 3 and 7°N, which featured very high depth-integrated *Trichodesmium* abundance (up to 9.4×10^4^ colonies m^-2^), strong thermohaline stratification and low zooplankton δ^15^N (~2‰). Relative atmospheric N input was lowest south of the equatorial upwelling between 3 and 5°S (27%). Values in the Guinea Dome region and north of Cape Verde ranged between 45 and 50%, respectively. The microstructure-derived estimate of the vertical diffusive N flux in the equatorial region was about one order of magnitude higher than in any other area (approximately 8 mmol m^-2^ d^ 1^). At the same time, this region received considerable atmospheric N input (35% of total). In general, zooplankton δ^15^N and *Trichodesmium* abundance were closely correlated, indicating that N fixation is the major source of atmospheric N input. Although *Trichodesmium* is not the only N fixing organism, its abundance can be used with high confidence to estimate the relative atmospheric N input in the tropical Atlantic (r^2^ = 0.95). Estimates of absolute N fixation rates are two- to tenfold higher than incubation-derived rates reported for the same regions. Our approach integrates over large spatial and temporal scales and also quantifies fixed N released as dissolved inorganic and organic N. In a global analysis, it may thus help to close the gap in oceanic N budgets.

## Introduction

Substantial uncertainties remain in oceanic nitrogen (N) budgets, especially in the tropical North Atlantic, that continue to stimulate critical reevaluation of diazotroph contribution to marine productivity [1–3]. The marine N cycle is closely coupled to the global carbon budget via primary production. The availability of several elements may limit oceanic primary production (N, P, Fe, Si, etc.) with N being typically the most important limiting nutrient on biological timescales and in large areas of the world´s oceans [4, 5]. Therefore, N availability largely determines the oceans capacity to act as a source or sink of atmospheric CO_2_. Regenerated N or inorganic nutrients that have been recycled in the upper ocean can support a large fraction of total primary production, but new N inputs are required to compensate N losses from surface waters [4, 6]. N losses from surface waters are mainly due to export of particulate matter by sinking and active transport via vertical migration of consumers [5]. Pelagic microbial N loss processes require suboxic to anoxic conditions [7] and are therefore generally considered of minor importance in the tropical Atlantic, where water column O_2_ concentrations usually exceed 40 μmol kg^-1^ [8].

The principal sources providing bioavailable N in the euphotic zone in the open ocean are vertical diffusive flux due to diapycnal mixing [1, 9], biological N fixation [1, 9, 10, 11] and atmospheric deposition [12, 13]. Especially in warm, stratified, oligotrophic waters, the fixation of atmospheric N by a variety of diazotrophs (such as *Trichodesmium* spp., diatom-associated cyanobacteria or UCYN-A, [14, 15]) represents a major source of new N for biological production in the mixed layer. In the equatorial Atlantic region, the dominant role of vertical mixing for supplying nutrients to the euphotic zone has long been recognized (e.g. [16]). Both observations and models confirm elevated chlorophyll and phytoplankton concentrations to be present throughout most of the year (e.g. [17, 18]). Nevertheless, recent findings challenge the general notion that N fixation is low in upwelling regions. Sohm et al. found high rates of N fixation in or near the Benguela Upwelling System [19], and Subramaniam et al. reported elevated N fixation rates in the equatorial Atlantic during the upwelling period [20]. Studies in the subtropical North Atlantic have demonstrated that depth-integrated N fixation rates by *Trichodesmium* can exceed the estimated vertical diffusive flux of NO_3_^-^ locally [1, 9]. Nevertheless, estimates of N fixation and vertical diffusive N flux do not cover the N demand of new production in a study conducted in the subtropical Northeast Atlantic [1], potentially because vertical diffusive N flux, N fixation or both were underestimated or because wet and dry deposition of N were not taken into consideration when calculating the atmospheric N input. We here use a combination of a biogeochemical tracer quantifying the relative contribution of atmospheric N input and direct measurements of vertical diffusive N flux to provide estimates of the total atmospheric N input to the pelagic food web for the ETNA.

The distinct sources of nitrogen to the pelagic food web have characteristic δ^15^N signatures. Atmospheric N is defined to have a δ^15^N value of 0‰ and diazotroph N fixation produces isotopically depleted biomass with δ^15^N values as low as -1 to -2‰ [10, 11, 21]. Inorganic N compounds in dust have a slightly lower δ^15^N signal of about -3‰ [13], whereas deep water nitrate in the Atlantic has a δ^15^N signature of approximately 4.5‰ [1]. Therefore, atmospheric N input results in a much lower biomass δ^15^N than biomass fuelled by nutrient rich deep water. Trophic fractionation then results in a relative increase in the heavy isotope during the transfer of N to higher trophic levels [22]. High zooplankton δ^15^N values ranging approximately between 8 and 12‰ occur in (and close to) upwelling areas, where biological production is principally supported by vertical mixing and advection of nutrient rich subsurface water (e.g. in the California Current system [23] and in the Eastern Tropical Atlantic [24]), whereas low zooplankton δ^15^N values between 1 to 5‰ have been found when *Trichodesmium* as a conspicuous diazotroph was present in high abundances [10, 11, 21]. The δ^15^N signature can therefore be used to trace the release of atmospheric derived N into the marine food web. Similarly, the δ^13^C signal is a tracer of food web structure, but its global distribution (i.e. isoscape) also shows considerable latitudinal variation, with maximum values >-20‰ observed in the tropical oceans [25–27]. Particularly heavy δ^13^C values around -13‰ were reported in *Trichodesmium* [28, 29] and hence the upper margin of the δ^13^C isoscape may be determined by N fixation. This would require exclusive *Trichodesmium* grazers like *Macrosetella gracilis* and *Miracia efferata* to have a fixed δ^13^C signature and that δ^13^C and δ^15^N of these organisms could be used to pinpoint the isotopic baseline of C and N entering the food web via N fixation.

In this study, the differential impact of atmospheric derived versus upwelled inorganic nitrogen to the food web of the tropical Atlantic was assessed. Atmospheric derived N is here defined as the sum of wet and dry deposition via dust and rain, as well as N fixation by diazotrophs. The vertical diffusive N flux was estimated from nutrient profiles, the ocean’s stratification, and concurrently collected microstructure shear data. Stable nitrogen isotopic signatures of zooplankton were used to estimate the relative input of atmospheric N to the surface waters. Estimates of vertical diffusive N flux and relative atmospheric input were then combined to yield absolute estimates of atmospheric N input. Where possible, estimates of wet and dry deposition from literature data were used to also estimate absolute N fixation for a given area. *Trichodesmium* abundance was determined using an Underwater Vision Profiler 5 to test the hypothesis that *Trichodesmium* abundance can serve as an indicator of atmospheric N input in the tropical Atlantic and to calculate the potential N fixation rate of *Trichodesmium*.

## Material and Methods

**Fig 1:**
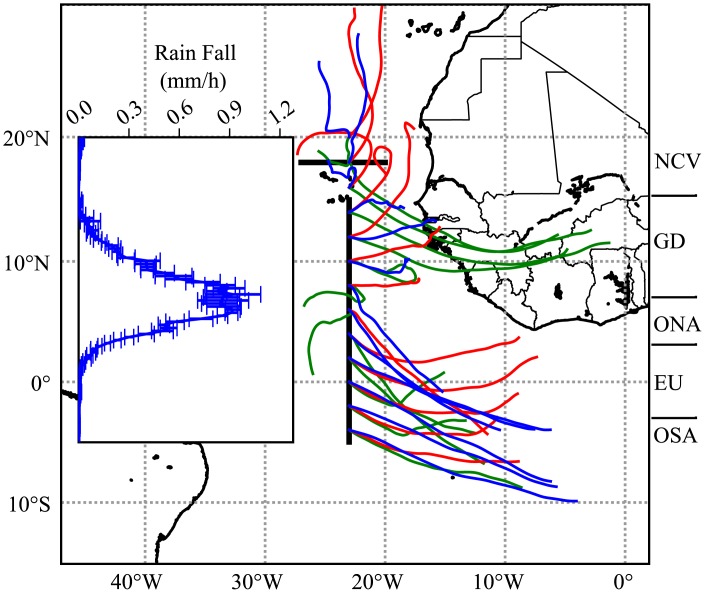
72-hour Hysplit back trajectories of air masses that reached positions along 23°W at 1000 m height for October 23 (green), November 8 (red) and November 16 (blue). The inset shows the average satellite rainfall (mm h^-1^) along 23°W within the longitude range 22°W to 24°W for November 2012. Black lines denote the sampled transects at 23°W and 18°N.

Sampling was conducted along a N-S transect from 15°N to 5°S at 23°W and along an E-W transect from 20 to 27°W at 18°N in the eastern tropical Atlantic (Fig 1) during R/V “Maria S. Merian” cruise MSM 22 (October 24—November 23, 2012). For sampling in the exclusive economic zone of Cape Verde, permission was granted by the Cape Verdean Ministry of Foreign Affairs. Work conducted in international waters did not require a specific permit and did not involve endangered or protected species. Satellite derived rainfall rates (NASA tropical rainfall measuring mission) and back trajectories of air masses for the region and time frame of our observations were downloaded from http://trmm.gsfc.nasa.gov/ and http://ready.arl.noaa.gov/HYSPLIT.php, respectively. Oceanographic observations were conducted using a 24-niskin bottle rosette with a Seabird SBE 11plus CTD equipped with a Dr. Haardt fluorescence probe and an Underwater Vision Profiler 5 (UVP 5, serial number 001). The fluorescence probe was cross-calibrated with regularly conducted chl-*a* measurements. Chl-*a* samples were 0.2-mm filtered (25 mm Whatman GF/F), the filters frozen at -80°C for over 5 h, extracted in 90% acetone and measured against a blank in a Turner Trilogy fluorometer calibrated with a chl-*a* standard dilution series. *Trichodesmium* distribution and abundance were quantified at 111 stations with the UVP5. This imaging tool allows *in situ* quantification of particles >60 μm and plankton >500 μm with high vertical resolution [30, 31]. Thumbnails of all objects > 500 μm were extracted using the ZooProcess software [32]. Imaged *Trichodesmium* were identified by a computer-assisted method [32] and the identification validated by experts. The observed volume of each image was 0.93 L. On average 11.6 (±3.09) images were recorded per m depth and the mean sampling volume for the upper 200 m of the water column was 2.16 m^3^.

Water column sampling was carried out at 25 stations. Samples for dissolved inorganic macronutrients (NO_3_^-^ + NO_2_^-^, PO_4_^3-^) were taken at eight depths within the upper 300 m (fixed depths 250, 150, 100, 80, 60, 40, 20, and 10 m), frozen at -80°C and stored at -20°C until later analysis in the home laboratory. Dissolved water column nutrients (NO_x_, PO_4_) were measured according to Grasshoff using a Quaatro autoanalyzer [33]. Depth (Z_50_) and thickness (H) of nitracline and phosphocline was determined following Hauss et al. by fitting sigmoid regressions of NO_3_^-^ and PO_4_^3-^ concentrations as a function of depth down to 150 m [24].

Zooplankton was collected with vertical tows of a 200 μm WP2 plankton net from 100 m to the surface and a number of widely distributed and frequently occurring species were chosen to represent four distinct trophic groups and sampled when available [34–36]. They comprised epipelagic copepods and juvenile euphausiids (Table 1). Individuals were identified, rinsed with distilled water, transferred into pre-weighed tin cups (5x9 mm, Hekatech), dried at 50°C for at least 48 hrs, weighed and prepared for elemental analysis of carbon and nitrogen amounts as well as their stable isotope ratios (δ^13^C and δ^15^N). See S1 Table for a complete summary of samples. Due to their small size, *Macrosetella gracilis* were collected on precombusted GF/F filters and packed into a tin capsule. Analysis was conducted as gas chromatography-combustion-isotope ratio mass spectrometry (GC/C/IRMS) at the UC Davis stable isotope facility (California, USA). Stable isotope ratios are reported with reference to a standard and expressed in parts per mil (‰) according to the formula: $\delta^{H}X\;=\;\left[\left(R_{SAMPLE}/\;R_{STANDARD}\right)\right]\;*1000$, where X is the respective element, H gives the heavy isotope mass of that element, and R is the ratio of the heavy to the light isotope [22].

**Table 1:** Zooplankton species analyzed in this study pooled by major feeding types according to references [34–36].

Feeding category	Zooplankton species
	**Copepod species**
Carnivore	*Candacia* sp.
	*Euchaeta marina*
*Trichodesmium*-grazer	*Macrosetella gracilis*
	*Miracia efferata*
Omnivore	*Pontella* sp.
	*Scolecithrix danae*
	*Undinula vulgaris*
	**Euphausiids**
Planktonic filter-feeder; omnivore	juveniles (mainly *Euphausia gibboides* and *Thysanopoda tricuspidata*)

A multiple linear regression model with zooplankton δ^15^N depending on depth of nitracline (Z_50N_), nitracline thickness (H_N_), and the difference between phosphocline and nitracline thickness (H_P_-H_N_) and all significant interactions was used to predict zooplankton δ^15^N for omnivore and carnivore epipelagic copepods as these groups could be sampled throughout the investigation area. Backward stepwise model simplification was used to identify significant predictors. Since the intercept of the multiple regression represents maximum δ^15^N under strong upwelling conditions (i.e. when vertical diffusive N flux providing 100% of N available to biological production), it was used as δ^15^N_Ref_ to calculate atmospheric contribution to zooplankton biomass (%N_Atm_). We applied the simple isotopic mixing model introduced by Montoya et al. [10]: $\%{\text{N}}_{\text{Atm}}=100*\left(\frac{\delta^{15}{\text{N}}_{\text{Zpl}}-\delta^{15}{\text{N}}_{\text{Ref}}}{\delta^{15}{\text{N}}_{\text{Atm}}-\delta^{15}{\text{N}}_{\text{Ref}}}\right)$, where δ^15^N_Ref_ is a baseline reference δ^15^N for zooplankton exclusively consuming NO_3_-fuelled POM and δ^15^N_Atm_ is a baseline value for atmospheric inputs (via dust deposition and diazotrophy). δ^15^N_Atm_ was assumed to be -2‰, reflecting the mean isotopic signature measured for diazotrophs and dust and therefore providing estimates of atmospheric contribution to zooplankton biomass [10, 13]. To explore the relationship between *Trichodesmium* abundance and the contribution of atmospheric N input to zooplankton biomass, we excluded the station where an anticyclonic mode-water eddy was sampled (18°N 20°W).

Microstructure shear and temperature profiles were collected using a loosely-tethered profiler (MSS 90D-II) manufactured by Sea&Sun Technology [37]. The profiler was equipped with two shear sensors (airfoil), a fast temperature sensor (FP07), an acceleration sensor, tilt sensors and standard CTD sensors. It was adjusted to descent at 0.5–0.6 ms^-1^. Two to five repeat profiles were collected following a CTD profile at each station from the surface to down to 1000 m depth (S2 Table).

High-frequency shear fluctuations measured by the airfoils were used to estimate the local dissipation rate of turbulent kinetic energy (ε). Wavenumber spectra were calculated from one-second ensembles of shear data (1024 individual measurements). ε was then determined by integrating the shear spectrum using the relationship for isotropic turbulence

ε=7.5μ(∂u∂z)¯2=7.5μ(∫kminkmaxEdu′dz(k)dk),

where μ is the dynamic viscosity of seawater, ∂u/∂z the vertical shear of horizontal velocity fluctuations, and $E_{du^{\prime }/dz}(k)$ the shear wavenumber spectrum. The lower wavenumber k_min_ was set to 3 cpm while the upper cutoff number k_max_ was varied by iteration between a maximum value of 30 cpm and a minimum value of 14 cpm when dissipation was low [38]. The loss of variance due to incomplete integration was compensated by extrapolating the observed spectrum in the neglected wavenumber band using the theoretical Nasmyth spectrum [39]. Similarly, loss of variance resulting from spatial averaging due to the finite size of the sensor tip was corrected following Macoun & Lueck [40]. Turbulent eddy diffusivities (K_ρ_) were calculated from ε and the buoyancy frequency (N), as K_ρ_ = ΓεN^-2^ [41]. Mixing efficiency, Γ, was set to 0.2. Stratification (N^2^ = g/ρ_o_∙dρ/dz–g^2^∙c^-2^, g—earth gravity, dρ/dz–vertical gradient of potential density, ρ_o_—reference potential density, c–speed of sound) was calculated from the CTD data using the adiabatic levelling method [42]. Overall the same procedure as described by Schafstall et al. was used [38]. The upward flux F of NO_3_^-^ + NO_2_^-^ across the nitracline (Z_50N_) due to turbulent mixing was calculated by multiplying the eddy diffusivity with the average vertical nitrate and nitrite concentration gradient between 25 and 75% of the subsurface concentration, F_NO3- + NO2-_ = K_ρ_ d(NO_3-_+NO_2-_)/dz.

Absolute atmospheric N input (wet and dry deposition, as well as N fixation) was calculated for each distinct oceanographic area as (diffusive N flux / % diffusive N flux)* % atmospheric N.

## Results

### Rainfall rates and air mass back trajectories

Satellite derived rainfall rates during the cruise along the 23°W section were high between 3 and 7°N and almost zero within the remaining sampling area (Fig 1). Backtracking of air masses from 1000 m height above the 23°W transect shows that stations south of 7°N were influenced by southeasterly winds stemming from the open Southeast Atlantic. Stations north of 7°N were influenced by northeasterly winds from the Sahara and Sahel region (Fig 1). Back trajectories for 0 and 500 m height were similar (data not shown).

### Hydrography and water column biogeochemistry

**Fig 2:**
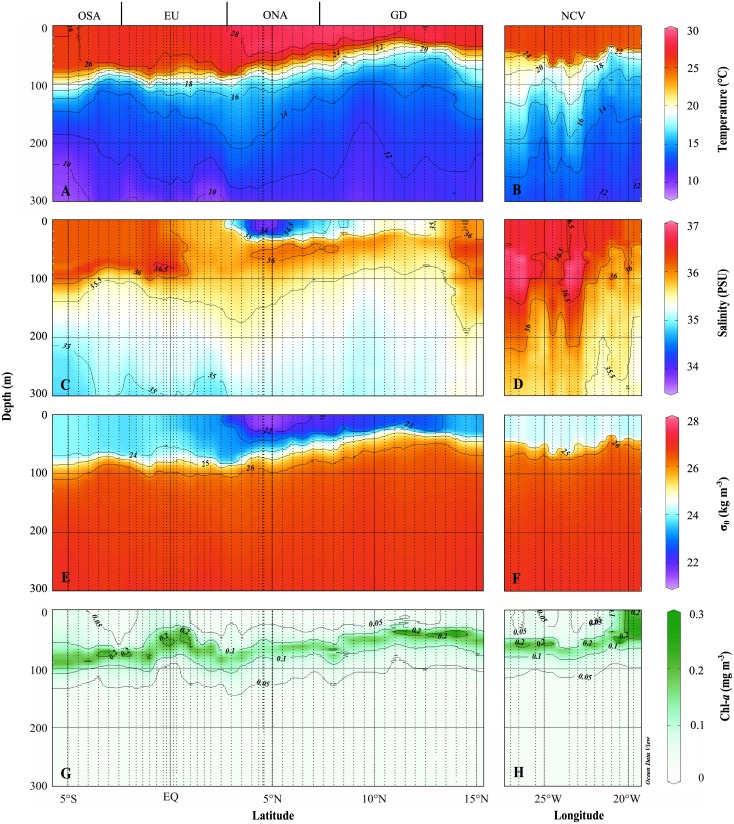
Sections of temperature (°C), salinity (PSU), potential density anomaly σ_θ_ (kg m^-3^) and chlorophyll-*a* (mg m^-3^) in the upper 300 m of the 23°W transect (A, C, E, G) and the 18°N transect (B, D, F, G), respectively.

Stations were grouped according to the following oceanographic areas: 3–5°S—oligotrophic South Atlantic (OSA), 3°S-3°N—equatorial upwelling region influenced by strong diapycnal mixing (EU), 3–7°N—oligotrophic North Atlantic (ONA), 7–15°N—Guinea Dome (GD), along 18°N—north of Cape Verde (NCV; Fig 2). South of approximately 3°S, the water column was highly stratified, but lacked superficial fresher water. Around the equator (3°S-3°N), a comparatively shallow and intense chl-*a* maximum and elevated vertical shear of horizontal velocity were observed due to the presence of the eastward Equatorial Undercurrent and westward South Equatorial Current (not shown). Between 3°N and 7°N, the water column was highly stratified, featuring a superficial “lens” of very low salinity and a deep chl-*a* maximum. In the GD region, the pycnocline was considerably shallower than in the southern portion of the transect, and the chl-*a* maximum was approximately as shallow and intense as in the EU. Along the 18°N transect (Fig 2, right panels), the deep chl-*a* maximum was generally shallower than at the 23°W transect and shoaling towards the eastern margin (Fig 2H). Within an anticyclonic mode water eddy at 19°40'W identified from shipboard ADCP data (not shown) the chl-*a* maximum extended to the surface.

### *Trichodesmium* distribution

**Fig 3:**
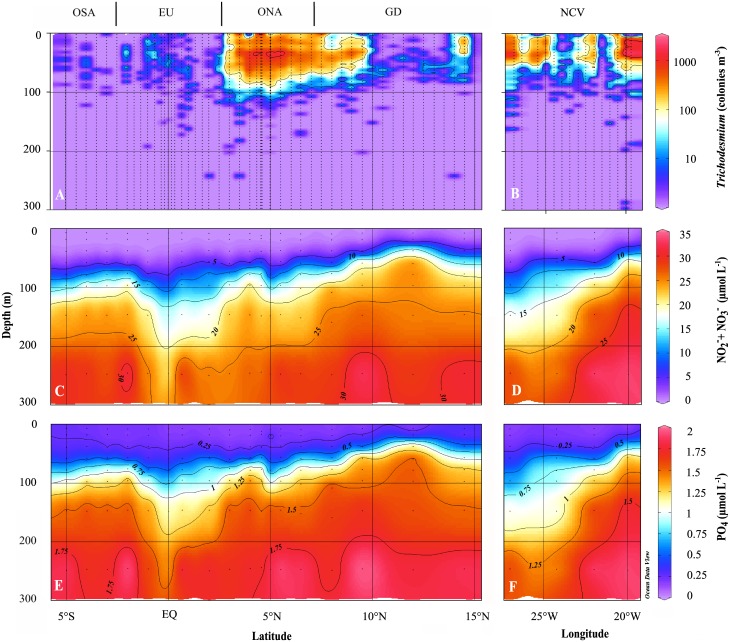
Sections of *Trichodesmium* abundance, NOx (NO_2_^-^+NO_3_^-^) and PO_4_^3-^ in the upper 300m of the 23°W transect (A, C, E) and the 18°N transect (B, D, F), respectively.

Along 23°W, a pronounced *Trichodesmium* bloom around 5°N extended to a depth of about 80 m with a clear peak around 40 m (Fig 3A). Water column integrated areal abundances of up to 9.4 × 10^4^ colonies m^-2^ were observed in this area. North of 10°N and in the equatorial region, abundance was lower but *Trichodesmium* was present in all profiles. South of 2°S only few colonies were observed, with some profiles being entirely void of *Trichodesmium*. *Trichodesmium* abundance on the 18°N transect was highly variable and the most conspicuous peak with up to 5.5 x 10^4^ colonies m^-2^ was found within the anticyclonic mode-water eddy at the easternmost station at 30 to 35 m depth (Fig 3B).

### Nutrient distribution

**Fig 4:**
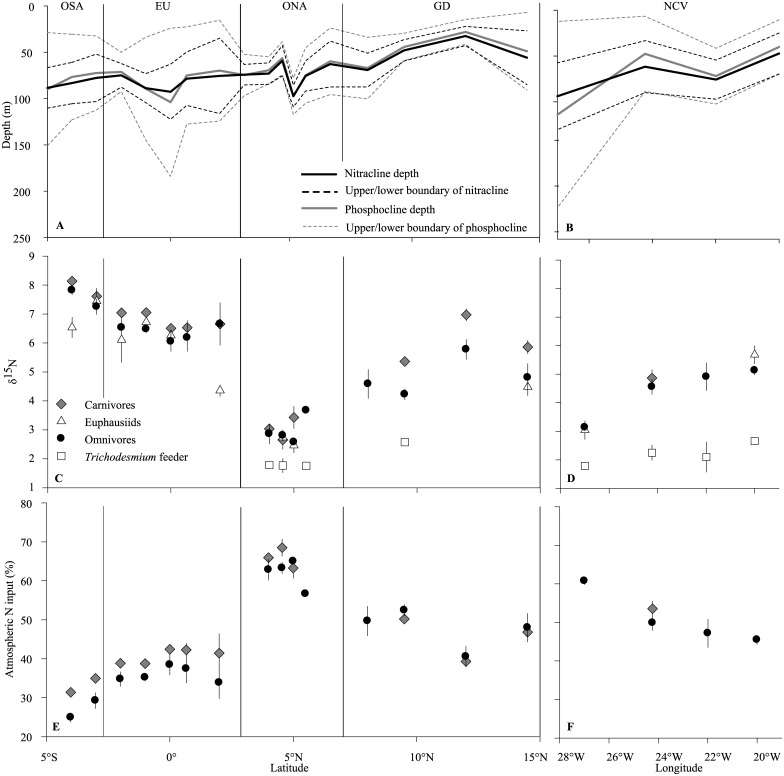
Spatial distribution of nitracline depth, nitracline thickness, phosphocline depth and phosphocline thickness (A, B), mean (±SD) zooplankton δ^15^N (C, D), and mean (±SD) relative contribution (%) of atmospheric input to zooplankton biomass (E, F) along 23°W and 18°N, respectively.

The spatial distribution of macronutrients was closely related to pycnocline depth (Figs 2 and 3). Mean near-surface (10m) concentrations of dissolved inorganic N (combined NO_2_^-^/NO_3_^-^) often reached the detection limit of 0.004 μmol L^-1^. North of about 7°N along the 23°W transect, a shallow nitracline with minimum depth of about 20 m was observed. Between 3 and 7°N, the nitracline was generally deeper and characterized by steep vertical gradients. At the equator, the vertical gradient was less sharp compared to off-equatorial locations and nutrient depletion reached deep into the water column. Along the 18°N transect, the nitracline ascended from west to east and its vertical extension decreased concomitantly (Figs 3 and 4). Mean near-surface values of dissolved inorganic phosphate (DIP) were 0.18 ±0.045 μmol L^-1^ and 0.14 ±0.021 μmol L^-1^ for the 23°W and 18°N transect, respectively (Figs 3 and 4). Along both transects, nitracline and phosphocline depths were highly correlated (0.96 cor, *p*<0.0001, Pearson`s test), with the nitracline being below the phosphocline with the exception of two stations.

### Stable isotopes in zooplankton

Along the 23°W transect, the zooplankton δ^15^N values varied widely and ranged from approximately 1.5‰ in *Trichodesmium* feeders to values exceeding 8‰ in carnivores at some stations. *Trichodesmium* grazers *Macrosetella gracilis* and *Miracia efferata* were only found at stations where *Trichodesmium* was present. Lowest zooplankton δ^15^N values were found around 5°N and increased toward north and south, with a peak at the southernmost station sampled (Fig 4C). Along the 18°N transect, δ^15^N values increased towards the east for all species, except in presumable *Trichodesmium* feeders with values as low as 2.3(±0.29)‰ throughout the entire transect (Fig 4D). At most stations, δ^15^N values were highest in carnivores. Lowest values were observed in *Trichodesmium* feeders (if present). δ^15^N values of omnivores and euphausiids mostly ranged in between those extremes, but no consistent order among them was evident across stations.

**Fig 5:**
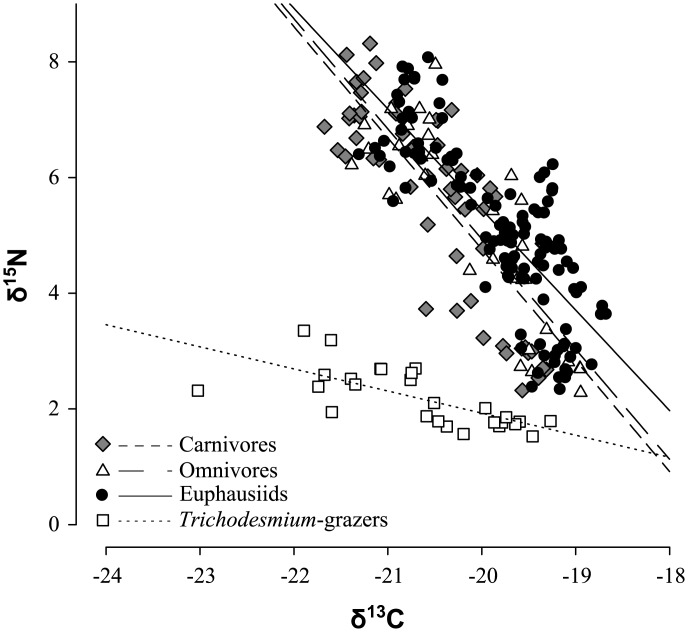
Negative linear relationship between individual bulk δ^15^N and δ^13^C values of zooplankton functional groups (*Trichodesmium*-grazer, carnivore, omnivore and euphausiids).

Zooplankton δ^13^C values ranged from a minimum of -23.0‰ in *Trichodesmium* feeders to -18.7‰ in omnivore species (Fig 5). Spatial differences in zooplankton δ^13^C were inversely related to δ^15^N values, with low values in the equatorial region and in the northern part of the 23°W transect that contrasted a peak of high values at about 5°N. At all stations present, δ^13^C values were lowest in *Trichodesmium* grazers. Among feeding types, δ^15^N and δ^13^C were negatively correlated (Fig 5). The slope of the corresponding linear regression was -0.38(±0.08) in presumable direct consumers of diazotroph biomass (*M*. *gracilis* and *M*. *efferata*), which was significantly different (ANCOVA, α = 0.05) from that in all other groups, with values of -1.9(±0.1), -1.7(±0.1) and -1.9(±0.2) for carnivores, omnivores and euphausiids, respectively.

### Diffusive NO_3_-flux

Microstructure shear-derived estimates of diffusive NO_3_-flux into the mixed layer were highly variable among individual stations, ranging between 50 and 23000 μmol m^-2^ d^-1^.This variability results from sporadic occurrence of elevated turbulence in the upper thermocline due to breaking of internal waves or shear instability of the zonal equatorial currents in the case of the EU region (see S1 Fig for eddy diffusivity vertical and latitudinal distribution). Turbulent dissipation rates were elevated by up to 4 orders of magnitude during an active mixing event. Due to elevated turbulence in the upper thermocline of the EU region and despite the weak vertical dissolved inorganic N gradient (Fig 3), the average diffusive flux was with 8200 μmol m^-2^ d^-1^ about one order of magnitude higher than in all other considered regions (Table 2). Average eddy diffusivities here were between six and twentyfold higher. In all other areas, eddy diffusivities were close to the background value of 1x10^-5^ m^2^s^-1^ and vertical diffusive N fluxes varied between 400 and 1000 μmol m^-2^ d^-1^.

**Table 2:** Regional estimates of daily new N input to the near surface layer based upon nutrient profiles, microstructure-derived turbulence, and % atmospheric contribution derived from stable isotope analysis. *n* represents the number of stations included in the respective region.

Area	relative atmospheric N input % (±SD) *n*			mean eddy diffusivity (K_ρ_) m^2^ s^-1^	diffusive NO_3_ fluxμmol m^-2^ d^-1^ (90% CI) *n*			atmospheric N input μmol m^-2^ d^-1^
OSA (3–5°S)	27.0	(±3.0)	2	2.7x10^-5^	1404	(302–2505)	3	520
EU (3°S-3°N)	35.1	(±1.0)	5	16.8x10^-5^	8208	(3456–14860)	5	4445
ONA (3–7°N)	61.4	(±0.7)	3	1.1x10^-5^	691	(250–1607)	5	1097
GD (7–15°N)	44.6	(±5.1)	5	0.8x10^-5^	505	(371–752)	3	407
NCV (17.6–18°N)	49.5	(±6.6)	5	2.0x10^-5^	1015	(234–2693)	3	994

### Contribution of atmospheric N input

For both omnivores and carnivores, δ^15^N was significantly negatively correlated with nitracline depth Z_50N_, nitracline thickness H_N_, and with the interaction H_N_:H_P_-H_N_ (H_P_: phosphocline thickness, Table 3). δ^15^N was significantly positively correlated with H_P_-H_N_ and with the interaction Z_50N_:H_N_. The remaining interactions were not significant in the model. The intercept of multiple linear regression was lower for omnivores (11.08±0.95), where the model explained 57% of the variability found, than for carnivores (12.78±1.76), where the model accounts for 51% of the variability. Overall δ^15^N of all zooplankton samples was significantly negatively correlated with depth-integrated *Trichodesmium* abundance (p<0.0001).

**Table 3:** Multiple linear regression parameters B (±standard error SE) of δ^15^N as a function of nitracline depth (Z_50N_), nitracline thickness (H_N_) and phosphocline thickness (H_P_).

	**Feeding category**	
	Omnivore	Carnivore
****Adjusted r**^**2**^**	0.57	0.51
**Intercept**	11.08(±0.95)***	12.78(±1.76)***
****B Z**_**50N**_**(±SE**_**B**_**)****	-0.15(±0.02)***	-0.15(±0.027)***
****B H**_**N**_**(±SE**_**B**_**)****	-0.18(±0.03)***	-0.23(±0.059)***
****B H**_**p**_**-H**_**N**_**(±SE**_**B**_**)****	0.13(±0.01)***	0.12(±0.024)***
****B Z**_**50N**_**: H**_**N**_**(±SE**_**B**_**)****	0.0041(±0.00048)***	0.0043(±0.00092)***
****BZ**_**50N**_**:H**_**p**_**-H**_**N**_**(±SE**_**B**_**)****		
****B H**_**N**_**: H**_**p**_**-H**_**N**_**(±SE**_**B**_**)****	-0.0026(±0.00022)***	-0.0024(±0.00056)***
****BZ**_**50N**_**:H**_**N**_**:H**_**p**_**-H**_**N**_**(±SE**_**B**_**)****		

**Fig 6:**
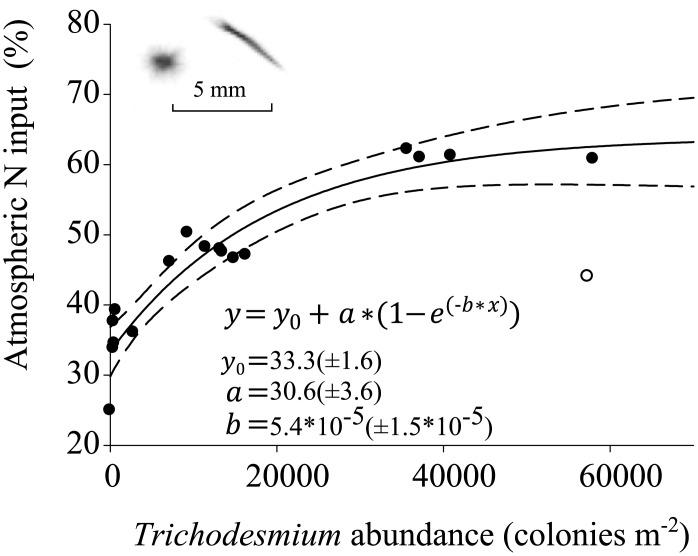
Contribution of calculated atmospheric N input (%) to zooplankton biomass as a function of integrated *Trichodesmium* abundance (colonies m^-2^). The station at 18°N, 19°41’W within the eddy (denoted with an empty circle) was excluded from the analysis. The inset shows UVP5 example images of Trichodesmium puff and tuft, respectively.

Relative contribution of atmospheric N input (wet and dry deposition, as well as N fixation) as calculated with δ^15^N of zooplankton biomass ranged from 23% in the southern part of the 23°W transect to a maximum of 71% around 5°N (Fig 4E). Atmospheric N input estimates for omnivores and carnivores where always closely related. Relative contribution of atmospheric N input was minimal south of the equator, peaked at about 5°N and slightly declined again further towards the north. Along 18°N, the significance of atmospheric N input decreased from west to east for omnivores (Fig 4F). Atmospheric N input to the marine foodweb was described as a nonlinear function of depth-integrated *Trichodesmium* abundance (r^2^ = 0.95, Fig 6).

## Discussion

The aim of this study was to estimate the relative contribution of new N to the pelagic food web by evaluating the vertical diffusive nitrate flux and the atmospheric input (the sum of wet and dry deposition, as well as N fixation). This was achieved by combining observed parameters varying on very different spatial and temporal scales. Measurements of the vertical distribution of macronutrients and the turbulence in the thermocline were combined to calculate associated vertical diffusive nitrate fluxes [38]. The isotopic composition of mesozooplankton body tissue was used to estimate relative atmospheric N [10, 11, 24]. At the smallest spatial and temporal scales of the parameter space, microstructure profiles represent a momentary snapshot of the ocean [43]. At the largest scales, isotopic signatures of different zooplankton ecotypes represent time-integrating *in situ* tracers of the biological processing of organic compounds in the water column [22]. Hence, the large variations in zooplankton nitrogen isotopes we found along the 23°W and 18°N transects indicate major changes in the source of nutrients. Observations of *Trichodesmium* abundance support this notion. Combining these methods to quantify the sources of nitrogen to the pelagic food web has the potential to provide a holistic view of global nitrogen budgets [10, 11].

The location of the deep chl-*a* maximum and the nutriclines mostly coincides with the pycnocline and is located between 50 and 90 m depth. Shallowest locations of these features are within the EU and GD region, albeit with large differences in vertical mixing due to different local physical processes: the diffusive nutrient flux is more than an order of magnitude higher in the equatorial upwelling region compared to the open ocean upwelling region, i.e. the GD. The shallow pycnocline in the GD is related to the positive wind stress curl in this region causing upward velocities due to Ekman divergence and a shoaling of the thermocline. At the equator, dynamic instabilities within the current system (eastward Equatorial Undercurrent and westward South Equatorial current) superimposed on the mean vertical shear flow enhance turbulence generation and thus diapycnal mixing in the upper thermocline (e.g. [44, 45]), resulting in a low vertical nutrient gradient, but a comparatively deep nutricline. Finally, the shoaling of the pycnocline in the mode water eddy is a consequence of the geostrophic adjustment due to the rotational velocities within the eddy. The nitracline was generally deeper and thinner than the phosphocline, suggesting that non-diazotroph primary production in the euphotic zone was first-of-all limited by N rather than P.

It is generally acknowledged that spatial differences in diffusive N flux are largely due to the high variability in eddy diffusivity rather than NO_3_-gradients [1]. The average K_ρ_ values determined in this study, despite being a single realization in time and space, are consistent with previously reported eddy diffusivities in the respective regions. In the upper thermocline away from the equator, our K_ρ_ estimates are in the range of 0.7-2x10^-5^ m^2^s^-1^. This is in agreement with reported K_ρ_ estimates for ONA and GD region at similar longitudes of 1x10^-5^ m^2^s^-1^ from multi-cruise microstructure observations [46, 47] as well as from a deliberate tracer release experiment [48]. At the equator, previously reported average K_ρ_ estimates vary on seasonal and annual time scales [44]. However, the overall magnitude of our K_ρ_ measurements again compares well to results from previous sampling campaigns: Hummels et al. reported K_ρ_ estimates 2-3x10^-4^m^2^s^-1^ for the equatorial region at 23°W from microstructure data collected during two November cruises [45], while Fernández-Castro et al. reported average K_ρ_ of 1.5x10^-4^ m^2^s^-1^ from a December cruise at the equator at about 30°W [47].

In general, diffusive N fluxes determined from observations strongly vary regionally and seasonally. With the exception of the EU region, our values from the tropical Atlantic range between 500 and 1400 μmol m^-2^ d^-1^. These fluxes are comparable to estimates (1040 μmol m^-2^ d^-1^) determined from observations at 18°N in the eastern tropical Atlantic during the winter season [38] and fall in the same range as values estimated by Capone et al. (46 to 736 μmol m^-2^ d^-1^), who used fixed K_ρ_ values of 1.1 and 3.7*10^-5^ m^2^ s^-1^ [1]. Similar diffusive N fluxes determined from measurements (35 to 1250 μmol m^-2^ d^-1^) were also reported from a N-S transect in the Atlantic conducted in April/May 2008 [49]. In that study, however, N fluxes determined at the equator were very low and in the subtropical Atlantic very high. It should be noted that these values were estimated in the mixed layer and their relevance is unclear. Much lower diffusive N fluxes ranging from 35–85 μmol m^-2^ d^-1^ were reported by Painter et al. in the subtropical North Atlantic [9]. On the other hand, strongly elevated diffusive N fluxes have been reported from upwelling and coastal regions. Schafstall et al. observed diffusive N fluxes exceeding 10000 μmol m^-2^ d^-1^ in the Mauritanian upwelling [38], accounting for a substantial part of the primary production in that region. These numbers are comparable to our results of 8200 μmol m^-2^ d^-1^ determined from the observations within the EU.

The spatial variability in NO_3_ flux does not fully explain the spatial variation in *Trichodesmium* abundance, indicating that not only the lack of NO_3_ supply defines the ecological niche of *Trichodesmium*, but other environmental constrains such as sea surface temperature, iron and P availability [5]. Likewise, the spatial variability in zooplankton δ^15^N of specific trophic levels is not solely linked to NO_3_ flux, providing evidence that major differences in the N source used for biological production occur, with regions either dominated by upwelled or atmospheric N input.

Natural abundance δ^15^N tracer techniques were used previously to estimate atmospheric N input into the pelagic food web. δ^15^N in surface NO_3_ proved difficult to measure at low concentrations [50]. Filtered seston [11] or size-fractionated zooplankton [10, 11, 21] represent a mixture of trophic levels and functional groups, hampering the estimation of an N_Ref_ value. Our approach to use individual zooplankton species to estimate the atmospheric N input to the marine foodweb at a defined trophic level and, thus, fixed N_Ref_ seems more straightforward. Furthermore, previous studies used the highest δ^15^N measurements of their respective cruises or reported in that area as a benchmark for δ^15^N_Ref_ to estimate relative contribution of upwelled N to total N input into the foodweb [10, 11, 21]. The occurrence of *Trichodesmium* in the OSA (albeit in low numbers and not in all profiles) indicates that even in this region N fixation occurred. Above described approach was therefore not applicable for us and we defined δ^15^N_Ref_ as the regression intercept of the stepwise multiple linear regression model following the approach of Hauss et al. [24]. This gives a theoretical maximum δ^15^N with upwelled nitrate as the only N source. While the cited authors [10, 11, 21] reported δ^15^N_Ref_ from 5.6‰ to 7.5‰, our results were perceivably higher with 11.1‰ and 12.8‰ for omnivores and carnivores, respectively. These values also coincide with those observed in upwelling regions [23, 24]. Using the model estimates, we found minimal atmospheric contribution (dry and wet deposition, as well as N fixation) south of the equator, still accounting for approximately 25 to 30% of secondary production. Thus, estimates of atmospheric input would be lower by this amount if the observed δ^15^N at the southernmost station would represent δ^15^N_Ref_. Atmospheric contribution was with >60% highest in the ONA region. Resulting absolute atmospheric input rates could be estimated at 0.4 mmol N m^-2^ d^-1^ in this region. Diazotroph organisms and atmospheric nutrient sources–in particular by wet deposition [51]—are therefore of particular importance in fuelling the pelagic food web in the oceanic desert of the ONA. Atmospheric contribution of N in the EU region was 30 to 40%, but due to the high diffusive N flux, this translates to 4 mmol N m^-2^ d^-1^ atmospheric input. From a biogeochemical point of view, the EU therefore represents a substantial source of atmospheric N in the Atlantic Ocean, probably due to the supply of iron and dissolved organic phosphorous via upwelling that is thought to foster N fixation [20].

Mirroring the spatial pattern of δ^15^N, zooplankton δ^13^C was low in the equatorial upwelling area and high in stratified oligotrophic waters around 5°N. At first glance, it seems therefore to represent a similarly meaningful proxy for N fixation. A global pattern of latitudinal gradients in organic δ^13^C, with a maximum of approximately -18‰ in the tropics and a minimum close to -30‰ in the southern Ocean, has been observed in particulate organic matter [25], zooplankton [27] and cephalopods [26] and was attributed to algal growth rates and water temperature [52]. The upper end member of this range could very well be defined by N fixation. This would, however, require the identification of a baseline signal. The δ^13^C signature of *Trichodesmium* ranges between approximately -15 and -12‰ [28, 29] and is higher than that of any other phytoplankton species described. Our data suggest that this signature is not fixed, because the range in δ^13^C in exclusive *Trichodesmium*-grazers is as wide as that of other zooplankton (-23 to -19‰) compared to a very narrow δ^15^N range. δ^13^C can therefore not serve as another tracer to estimate N fixation and the reason for the variability of δ^13^C in the observation region remains enigmatic.

The spatial and temporal association between wet and dry deposition and diazotroph blooms due to iron fertilization complicates the distinction of these two N sources (e.g. [5]), and the δ^15^N approach fails to discern between biologically fixed nitrogen and nitrate derived from wet and dry deposition. In the majority of studies, the contribution of atmospheric deposition relative to biological N fixation rates in the ETNA was considered to be small (e.g. [11, 21, 24]). Since direct soluble N input would potentially inhibit diazotroph competitiveness, and reported values of leachable total nitrogen (LTN) flux are comparatively low [53], the importance of Saharan dust pulses rather seems to lie in fertilization of diazotroph growth by iron and phosphorous supply [5]. Although the diversity of N fixing organisms in the region is high [3, 14, 15], high abundance of *Trichodesmium* likely indicate favorable conditions for N fixing organisms in general. δ^15^N in omni- and carnivore zooplankton were strongly negatively correlated with *Trichodesmium* abundance. Only at the eastern margin of the 18°N transect, high *Trichodesmium* abundance observed in the core of an anticyclonic mode-water eddy (see e.g. [54] for a characterization of such eddies) was related to a high zooplankton δ^15^N. This structure featured a comparatively shallow nutricline, high primary productivity as indicated by satellite ocean color, high particle load and a shallow O_2_ minimum (data not shown). It thus seemed to represent an aging core of oceanic productivity. Diazotrophs such as *Trichodesmium* apparently take advantage of residual macro- and micronutrients when non-diazotrophs begin to be nutrient limited in the upper mixed layer. As zooplankton have a lower tissue turnover and generation time than phytoplankton [55], a recent change in the N source from upwelling to N fixation in this transient feature might be reflected in the high *Trichodesmium* abundance, but not yet in an altered zooplankton δ^15^N. The otherwise tight correlation of *Trichodesmium* abundance with omni- and carnivore zooplankton δ^15^N results in a significant relationship of *Trichodesmium* abundance and atmospheric N input and also indicates that N fixation is probably the major atmospheric source of N. However, *Trichodesmium* abundance can never explain all of the atmospheric contribution (even if used as a proxy for all N fixing organisms), as this would translate into an observation of zero dust input and occurrence of *Trichodesmium* at the same time. These conditions are not expected to co-occur due to iron limitation, resulting in an asymptotic behavior of the curve describing the *Trichodesmium* impact on the total atmospheric contribution.

Comparing to literature values of wet and dry deposition, absolute N fixation can be estimated. HYSPLIT back trajectories show that OSA and EU are influenced by air masses from the Southeast, which are nearly aerosol free [51], while the areas ONA, GD and NCV receive dust-laden air from continental West Africa. TRMM rainfall estimates indicate that ONA receives considerable amounts of wet deposition, whereas nearly no rainfall occurred in the other observation regions. It has recently been suggested that the intertropical convergence zone (ITCZ) forms a “biogeochemical divide” of the subtropical Atlantic, washing atmospheric dust (the major dissolved iron source) into micronutrient-depleted surface waters [51]. This is in line with our observations along the 23°W transect, where the high rainfall rates and a superficial lens of fresher water coincided with a massive *Trichodesmium* bloom at 5°N and the highest relative contribution of >60% atmospheric N to zooplankton biomass. It is unlikely that terrigenous material from the western part of the basin (Amazon River Plume) contributes quantitatively to the low δ^15^N values at the ONA region, given that only a small fraction of the plume is bound eastward [56], that the impact of riverine input is very small compared to precipitation at this longitude ([56], their Fig 7), and that we directly observed both the *Trichodesmium* bloom and the highest rainfall at 5°N. This led us to the conclusion that the wet deposition in the ITCZ is the main driver of the isotope pattern. In consequence, the observation regions can be divided into three categories: i) OSA and EU with little to no wet and dry deposition during the observation time frame ii) ONA with strong wet deposition that can not be constrained by literature estimates and iii) GD and NCV with very little to no wet deposition during the observation time frame and an almost constant dry deposition that can be constrained by literature estimates. If we assume dust deposition in the OSA and EU areas to be close to zero, the atmospheric contribution of 27 to 35% in these areas would be entirely due to biological N fixation. Very high N fixation rates of up to 4 mmol m^-2^ d^-1^ result for the EU region. While some authors consider the atmospheric contribution, and in particular diazotrophy in the equatorial upwelling to be quite low [10, 11], a similar observation has been made by Mouriño-Carballido et al. [49]. Subramaniam et al. even found that N fixation rates around the equator were 2 to 7 times higher during an upwelling event than during non-upwelling conditions and conclude that upwelled waters rich in phosphate and iron promote diazotrophy [20]. As the ONA mainly receives wet deposition, of which the LTN flux is not known, the absolute N fixation input cannot be estimated for this region. At Cape Verde, little variation in LTN flux over the year was reported [53]. If we assume the reported mean annual value of 32.6 μmol m^-2^ d^-1^ LTN for the GD and NCV regions, N fixation would account for 350 and 950 μmol m^-2^ d^-1^ (Table 2), respectively. In the GD region at 23°W during the same season, a mean of 194 μmol m^-2^ d^-1^ was measured in incubation experiments [3].

If we assume a colony-specific N fixation rate by *Trichodesmium* of 4.4 nmol d^-1^ (which is the mean of the incubation studies summarized in [57],their Table 7), mean watercolumn N fixation by *Trichodesmium* based upon UVP5 colony counts would be 1.8, 6.7, 188.6, 21.7 and 69.1 μmol m^-2^ d^-1^ in OSA, EU, ONA, GD and NCV, respectively. Differences to our estimates of N fixation for OSA, EU, GD and NCV are likely due to the fact that *Trichodesmium* represents only a fraction of the diazotroph community [14,15]. Furthermore, we could only quantify colonies >500 μm in the water column. Smaller colonies and single trichomes, as well as accumulations of *Trichodesmium* at the surface [58] could not be quantified. The observed very close relation of atmospheric N input and *Trichodesmium* abundance indicates that UVP5 estimates of *Trichodesmium* abundance can serve as a very good indicator for the general existence of a niche for diazotrophs, but these abundance estimates cannot be used to quantify the total N fixation in an area.

In general, our indirect estimates of N fixation rates are two- to tenfold higher than rates measured with incubation methods in the same regions [3, 20, 49]. Part of this large discrepancy may be due to uncertainties in direct N fixation rate measurements, as handling of N fixing organisms during shipboard incubations may disrupt N fixation capacity. On the other hand, recent findings suggest bioavailable N compounds in commercial ^15^N_2_ gas used for incubations bias the measurement [59]. Furthermore, current techniques only estimate the amount of N fixed in the particulate matter fraction that is obtained after filtration of the incubation volume. Fixed N that is directly released again as dissolved organic or inorganic N during the incubation time is currently not measured in N fixation incubation experiments, but in the open ocean will ultimately be transferred into the pelagic food web via the bacterial loop and therefore needs to be considered. Our approach is incubation independent, integrates over large spatial and temporal scales and also quantifies fixed N lost via exudation and therefore might provide a more realistic overall estimates of N fixation for the OSA, EU, GD and NCV regions. In a global analysis, it may help to close the gap in oceanic N budgets [2, 3] as it yields N fixation estimates that are about 10-fold higher than incubation techniques in some regions. Nevertheless, a direct comparison of incubation and the here used tracer technique would benefit our understanding of the oceanic N cycle.

## Supporting Information

S1 Fig Eddy diffusivity (K_ρ_) section along 23°W (Panel A) and grouped by region (Panel B; colored crosses denote individual profiles, corresponding colored horizontal lines maximum NO_x_ gradient and black line region mean). Mixed layer data are omitted from plots.
(DOCX)

S1 Table Species and number of individuals sampled at each station (three replicates each). (DOCX)

S2 Table Number of stations and microstructure profiles used to compute regional mean values. (DOCX)
