# Supplementary material for: Nitrogen Fuelling of the Pelagic Food Web of the Tropical Atlantic
Source: PLoS One. 2015 Jun 22;10(6):e0131258. doi: 10.1371/journal.pone.0131258 (PMC4476781; doi:10.1371/journal.pone.0131258)

S1Fig. Eddy diffusivity (K_ρ_) section along 23°W (Panel A) and grouped by region (Panel B; colored crosses denote individual profiles, corresponding colored horizontal lines maximum NO_x_ gradient and black line region mean). Mixed layer data are omitted from plots.

**A**


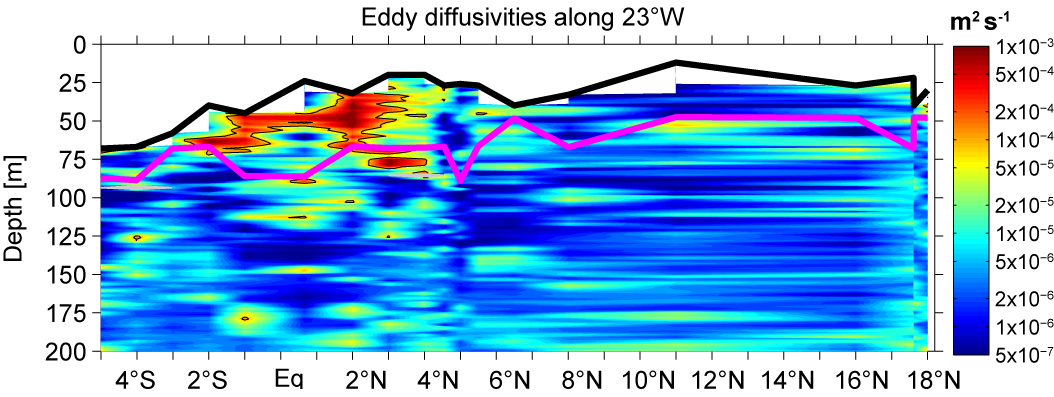


**B**


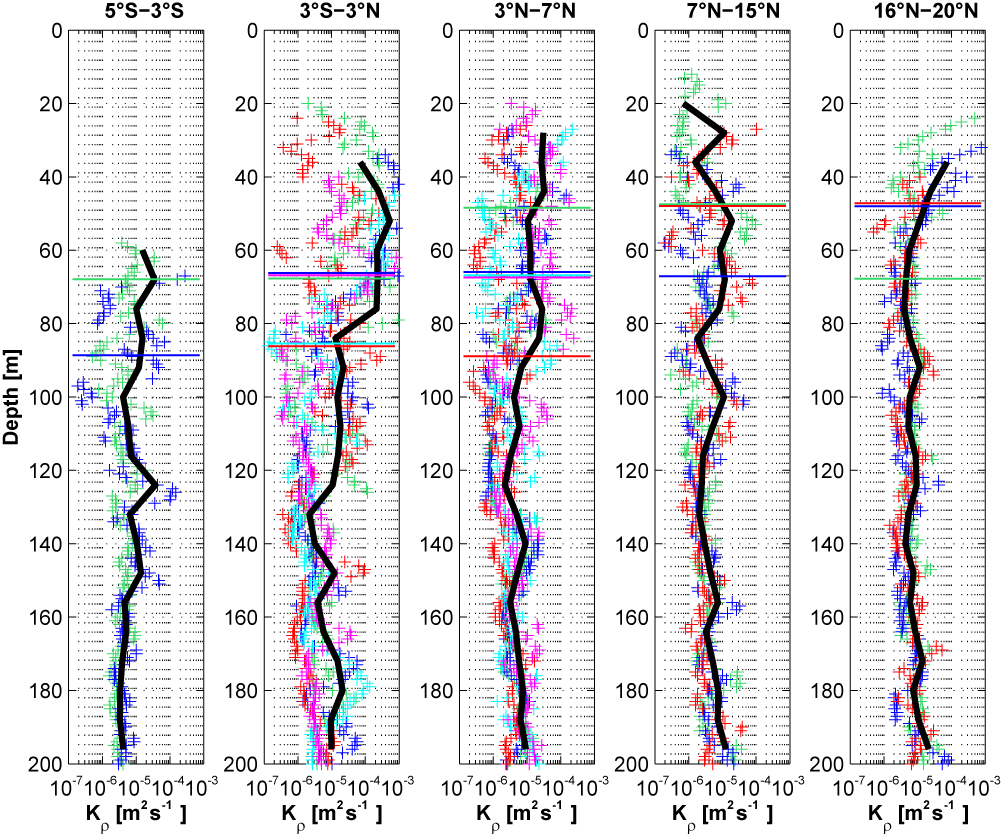

Supplement: S1 Fig — Mixed layer data are omitted from plots. (DOCX) [file pone.0131258.s001.docx]
