# Supplementary material for: Nitrogen Fuelling of the Pelagic Food Web of the Tropical Atlantic
Source: PLoS One. 2015 Jun 22;10(6):e0131258. doi: 10.1371/journal.pone.0131258 (PMC4476781; doi:10.1371/journal.pone.0131258)
Supplement: S1 Table — (DOCX) [file pone.0131258.s002.docx]

S1 Table. Species and number of individuals sampled at each station (three replicates each).

| CTD # | Latitude | Longitude | *U. vulgaris* | *E. marina* | *S. danae* | *Pontella* sp. | *Candacia* sp. | *M. gracilis* | *M. efferata* | *Euphausiids* |
| --- | --- | --- | --- | --- | --- | --- | --- | --- | --- | --- |
| 2 | 17°36.23’N | 24°14.59’W | - | - | 10-12 | - | 10 | ca. 40 | - | - |
| 9 | 10°59.58’N | 21°15.06’W | 8-9 | - | - | - | ­- | - | - | - |
| 13 | 8°01.00’N | 22°58.58’W | 9-10 | - | 5 | - | - | - | - | - |
| 20 | 5°29.59’N | 22°59.58’W | - | - | - | 10 | - | - | 40 | - |
| 24 | 5°00.02’N | 22°59.54’W | 4-5 | 3 | - | - | 4 | - | - | 5 |
| 30 | 4°33.02’N | 22°24.59’W | 4 | - | - | - | 4 | - | 20 | 5 |
| 32 | 4°00.00’N | 23°00.00’W | 4 | - | 5 | - | 8 | - | 20 | - |
| 40 | 2°00.00’N | 23°00.00’W | 5 | 7-9 | - | - | 5 | - | - | 5 |
| 44 | 0°39.58’N | 23°00.02’W | 5 | 9-10 | 4-5 | - | 5 | - | - | - |
| 48 | 0°00.13’N | 23°06.46’W | 5 | - | 5 | - | 4 | - | - | 5 |
| 53 | 0°59.56’S | 22°59.59’W | 5 | 5 | 4-5 | - | - | - | - | 4-6 |
| 58 | 1°59.58’S | 22°59.57’W | - | 4 | 5 | - | - | - | - | 3-4 |
| 60 | 3°00.39’S | 22°59.29’W | 5 | 5 | 9-10 | - | - | - | - | 4 |
| 65 | 4°00.40’N | 22°58.56’W | 5 | 5-6 | 5 | - | - | - | - | 4-6 |
| *722-2* | 5°56.79’S | 22°59.94’W | 5 | - | 5 | - | 5 | - | - | - |
| 74 | 9°30.06’N | 22°59.45’W | 5 | 4-5 | 5 | - | - | - | 17 | - |
| 82 | 12°00.00’N | 23°00.00’W | 5 | 5 | 5 | 4 | - | - | - | - |
| 89 | 14°29.58’N | 23°00.00’W | 5-6 | 5 | 5 | - | - | - | - | 3 |
| 93 | 15°59.59’N | 20°40.00’W | 5 | 5 | 5 | 4 | - | - | - | - |
| 95 | 18°00.05’N | 20°00.07’W | 5 | - | 5 | - | - | 45 | - | 3 |
| 99 | 18°00.34’N | 21°59.27’W | 4-5 | - | 5 | 2 | - | 27 | - | - |
| 104 | 17°37.00’N | 24°13.59’W | 5 | - | 5 | - | 3 | 35 | - | - |
| 111 | 18° 00.29'N | 26° 59.49'W | - | - | 5 | - | - | - | 26 | 4 |
