# Supplementary material for: Nitrogen Fuelling of the Pelagic Food Web of the Tropical Atlantic
Source: PLoS One. 2015 Jun 22;10(6):e0131258. doi: 10.1371/journal.pone.0131258 (PMC4476781; doi:10.1371/journal.pone.0131258)
Supplement: S2 Table — (DOCX) [file pone.0131258.s003.docx]

S2 Table. Number of stations and microstructure profiles used to compute regional mean values.

| **Latitude range** | **Latitude of Stations** | **CTD profile IDs** | **Number of microstructure**  **profiles** |
| --- | --- | --- | --- |
| 5°S-3°S | 5°S, 4°S, 3°S | 60, 63, 65 | 7 |
| 3°S-3°N | 3°S, 2°S, 1°S, 0.6°N, 2°N, 3°N | 36, 40, 44, 48, 53, 58 | 14 |
| 4°N-7°N | 4°N, 4.5°N, 5°N, 5.5°N, 6.5°N | 17, 20, 23, 30, 32 | 26 |
| 8°N-16°N | 8°N, 11°N, 16°N | 9, 13, 93 | 8 |
| >16°N | 17.6°N, 18°N, 17.6°N | 2, 95, 104 | 21 |
